# A novel “off-on” fluorescent probe for the detection of nickel ions and its clinical application

**DOI:** 10.3389/fbioe.2023.1261178

**Published:** 2023-09-18

**Authors:** Hua-Wei Yi, Xian-Mo Wang, Jia-Hao Wu, Chang-Li Zhang, Yi-Di Ding

**Affiliations:** ^1^ Laboratory Department, The First Affiliated Hospital of Yangtze University, Jingzhou, Hubei, China; ^2^ Laboratory Department, The First People’s Hospital of Jingzhou, Jingzhou, Hubei, China; ^3^ Department of Pharmacy, The First Affiliated Hospital of Yangtze University, Jingzhou, Hubei, China; ^4^ Department of Infectious Diseases, Tangdu Hospital, Air Force Medical University, Xi’an, Shaanxi, China

**Keywords:** nickel ions, fluorescent probe, off-on fluorescent probe, clinical application, sequence-specific cleavage

## Abstract

Nickel serves as an essential micronutrient for the human body, playing a vital role in various enzymatic processes. However, excessive nickel entering the environment can cause pollution and pose serious risks to animals, plants, and human health. High concentrations of nickel ions in the human body increase the risk of various diseases, highlighting the need for accurate measurement of nickel ions levels. In this study, we designed a sequence-specific cleavage probe for nickel (II) ion called SSC-Ni. Similar to the TaqMan probe, SSC-Ni is an off-on fluorescent probe with an exceptionally low background fluorescence signal. It exhibits high detection specificity, making it highly selective for nickel ions, and the detection limit of the probe towards Ni^2+^ is as low as 82 nM. The SSC-Ni probe can be utilized for convenient and cost-effective high-throughput quantitative detection of nickel ions in serum. Its user-friendly operation and affordability make it a practical solution. By addressing the lack of simple and effective nickel ion detection methods, this probe has the potential to contribute significantly to environmental monitoring and the protection of human health.

## 1 Introduction

Nickel, a widely used transition metal in industrial production, is also an essential micronutrient distributed throughout the human body. It mainly exists as divalent ions and plays a vital role in numerous enzymes (Genchi et al., 2020; Anchidin-Norocel et al., 2021). Nickel ions are found in various tissues such as the liver, kidney, spinal cord, brain, heart, cartilage, lung connective tissue, and skin (Afzali and Mohammadi, 2011; Rajivgandhi et al., 2021). However, excessive uptake of nickel ions can lead to various diseases, including asthma, acute pneumonitis, dermatitis, lung cancer, and sinus cancer (Kang et al., 2017). Meta-analysis has indicated a correlation between high serum nickel levels and breast cancer, suggesting nickel exposure as a risk factor (Yu and Zhang, 2017). Studies by Chiou et al. (2014) have also linked nickel accumulation in lung tissues to an increased risk of p53 mutation and lung carcinogenesis. Moreover, excessive nickel entering the environment can cause pollution, making it critical to monitor nickel ion levels in the environment, animals, and plants.

Traditional methods for detecting trace amounts of nickel include flame atomic absorption spectrometry (Lemes and Tarley, 2021; Rocha and Chemistry, 2021), inductively coupled plasma mass spectrometry (Musil et al., 2021; Xu et al., 2021), and flame photometry (de Oliveira et al., 2017; Barahoei et al., 2021). However, these methods often require expensive equipment and complex sample processing, limiting their practical application. In recent years, nickel ion-selective electrodes have been used for monitoring nickel ions (Abbaspour and Izadyar, 2001; Tashkhourian, 2015; Sheikh et al., 2017). Nevertheless, many of these electrodes have high detection limits, narrow working concentration ranges, and are susceptible to interference from various ions, such as H^+^, Na^+^, K^+^, Ba^2+^, Ag^+^, and Hg^2+^. Alternatively, fluorescent probes have gained the attention of their high sensitivity, selectivity, and ease of use in nicked ion detection (Song et al., 2021; Wang et al., 2022a; Wang et al., 2022b).

Building upon the work of Dang et al. (2019), who reported polypeptide sequences specifically cleaved by nickel ions. We designed a sequence-specific cleavage probe for nickel ions known as SSC-Ni. Inspired by the principles of the TaqMan probe (Cao et al., 2012), the SSC-Ni probe utilizes an “off-on” fluorescence mechanism by attaching a fluorescence quencher and fluorophore at both ends of the polypeptide sequences (Gly-Ser-His-His-Trp). To the authors’ knowledge, no fluorescent probes based on sequence-specific cleavage by nickel ions have been reported. This probe demonstrates high specificity in detecting nickel (II) ion, with a detection limit as low as 82 nM. Notably, the probe offers the advantages of simple operation and low cost. Additionally, we have conducted preliminary investigations into the application of the SSC-Ni probe for detecting nickel ions in the blood.

## 2 Materials and methods

### 2.1 Synthesis of fluorescent probe SSC-Ni

The amino acids sequence of the SSC-Ni probe is Gly-Ser-His-His-Trp (-GSHHW-). During the polypeptide chains synthesis, the Dabcyl group was introduced at the amino terminus and Lys (5-FAM) was introduced at the carboxyl terminus. The synthesis of probe SSC-Ni was completed by GL Biochem Ltd. (Shanghai, China).

### 2.2 Quality detection of fluorescent probe

HPLC was used to detect the purity of probe SSC-Ni. The C18 column with a volume of 10 μL was utilized, solvent A was 0.1% trifluoroacetic in 100% acetonitrile, solvent B was 0.1% trifluoroacetic in 100% water, the flow rate was 1.0 mL/min, and the detection wavelength was 220 nm. The molecular weight of the probe was detected with the Agilent-6125B mass spectrometer, and the theoretical molecular weight is 1,361.40 Da.

### 2.3 Degradation assay of probe SSC-Ni

Nickel ions with final concentrations of 1, 10, 100, and 1,000 μM were added to the 5 μM SSC-Ni probe under the reported buffer conditions (Dang et al., 2019), and the buffer for SSC-Ni probe in this study were 50 mM HEPES, 100 mM NaCl, and pH 8.2. Leave the samples at room temperature (∼25°C) for 12 h and observe the color change of the solution.

### 2.4 Fluorescence signal detection of probe

Different concentrations of nickel ions or other metal ions were added to the probe SSC-Ni, and the final concentration of the probe was 1 μM. The buffer condition was 50 mM HEPES 100 mM NaCl, and pH 8.2. Take 25 μL in a PCR tube and detect the real-time fluorescence intensity of FAM by qPCR instrument. The program setting of the PCR instrument included 60 cycles of hold at 37°C for 20 min, followed by data acquisition at 37°C for 30 s on AGS 4800 real-time PCR system (Bioanyu Technology Co., Ltd., Hangzhou, China).

### 2.5 Calculation of detection limit

The detection limit was calculated from the formula: DL = 3 × SD/k, where SD is the standard deviation of the blank solution, k is the slope of the calibration curve (Yu et al., 2016). In the probe solution without nickel ions, there was no significant change in fluorescence intensity, and low concentration of nickel ions can be used as a blank solution to calculate SD value (Zare et al., 2017). Ten times of fluorescence intensity curve of probe with 0.1 μM Ni^2+^ were test and SD could be calculated from fluorescence enhancement rate (slope). The linear regression curve of probe SSC-Ni was then fitted to fluorescence enhancement rate of nickel ions at different concentrations to calculate the slope k.

### 2.6 Nickel ions detection in blood samples

Four blood samples without anticoagulants were taken, and serum was obtained by centrifugation at 4,000 rpm for 5 min after the blood was fully coagulated. The probe SSC-Ni with a final concentration of 1 μM was added to the serum, or additional 1, 10 μM of Ni^2+^ were added. Meanwhile, serum without probe was used as a negative control, and the fluorescence signal of the FAM channel was detected by qPCR instrument at 37°C.

### 2.7 Data analysis

The qPCR instrument was used to monitor the degradation of the probe SSC-Ni by nickel ions in real-time. The raw data of fluorescence intensity was exported, then the background fluorescence signal was deducted, and finally the data was imported into the Origin2018 software for linear fitting and mapping. The relevant fitting parameters such as intercept, slope, and *R* ^2^ could be obtained.

## 3 Results

### 3.1 Design and detection of probe SSC-Ni

**FIGURE 1:**
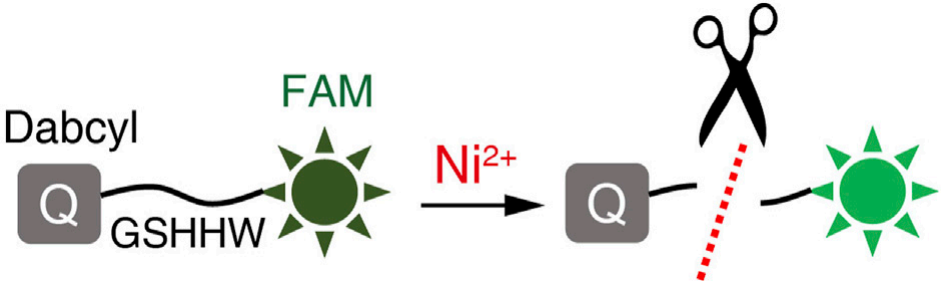
Schematic diagram of the probe SSC-Ni. The Dabcyl quenching group was attached at the amino end of the -GSHHW- sequence and the FAM fluorophore was attached at the carboxyl terminus. The fluorescence signal of FAM can be inhibited by Dabcyl. Nickel ions specifically cleave the -GSHHW- sequence and FAM fluorescence signals can be detected in the presence of appropriate excitation light.

Site-specific protein cleavage is essential for many protein production protocols, and the polypeptide sequence Gly-Ser-His-His-Trp (-GSHHW-) can be recognized and specifically cleaved by nickel ions, and the cleavage site of the -GSHHW- sequence is between G and S residues (Dang et al., 2019). Using the peptide chains *in vitro* synthesis method, we synthesized the -GSHHW- sequence and connected Dabcyl fluorescent quencher and FAM dye at its amino and carboxyl terminals, respectively. Thus, the probe SSC-Ni for detecting nickel ions was designed as shown in Figure 1. Nickel ions were able to degrade the -GSHHW- sequence and release the free FAM fluorophore so that the fluorescence signal can be detected.

**FIGURE 2:**
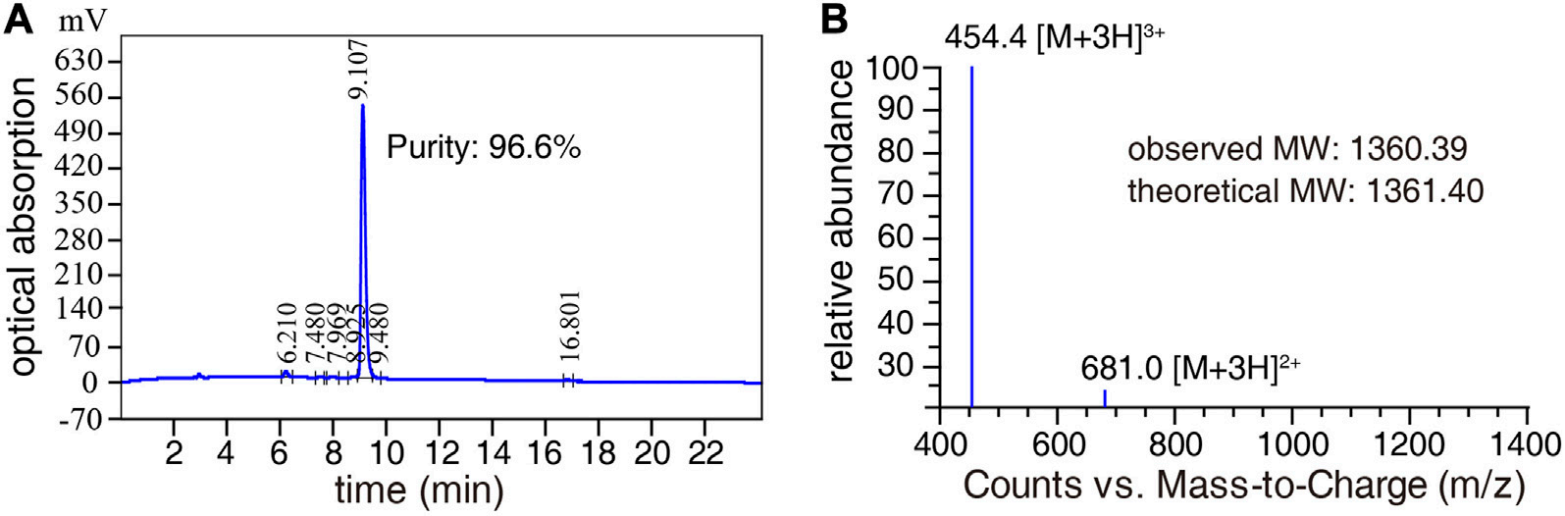
HPLC and mass spectrometry detection of SSC-Ni probe. **(A)** The purity of probe SSC-Ni was detected by HPLC, using a C18 column with a volume of 10 μL. The mobile phase flow rate was 1.0 mL/min and the detection wavelength was 220 nm. **(B)** The molecular weight of the probe SSC-Ni was detected by the Agilent-6125B mass spectrometer, and the molecular weight was measured as 1,360.39 Da.

Purity analysis of the SSC-Ni probe was performed using high-performance liquid chromatography (HPLC), as shown in Figure 2A. There was a high peak at the elution time of 9.107 min, and the proportion of this peak was as high as 96.6% through area integration, which indicates that the SSC-Ni probe has high purity. Meanwhile, we also used a mass spectrometer to detect probe SSC-Ni, as shown in Figure 2B, with an observed molecular weight of 1,360.39 Da, which is very close to the theoretical molecular weight.

### 3.2 Degradation of probe SSC-Ni by nickel ions

**FIGURE 3:**
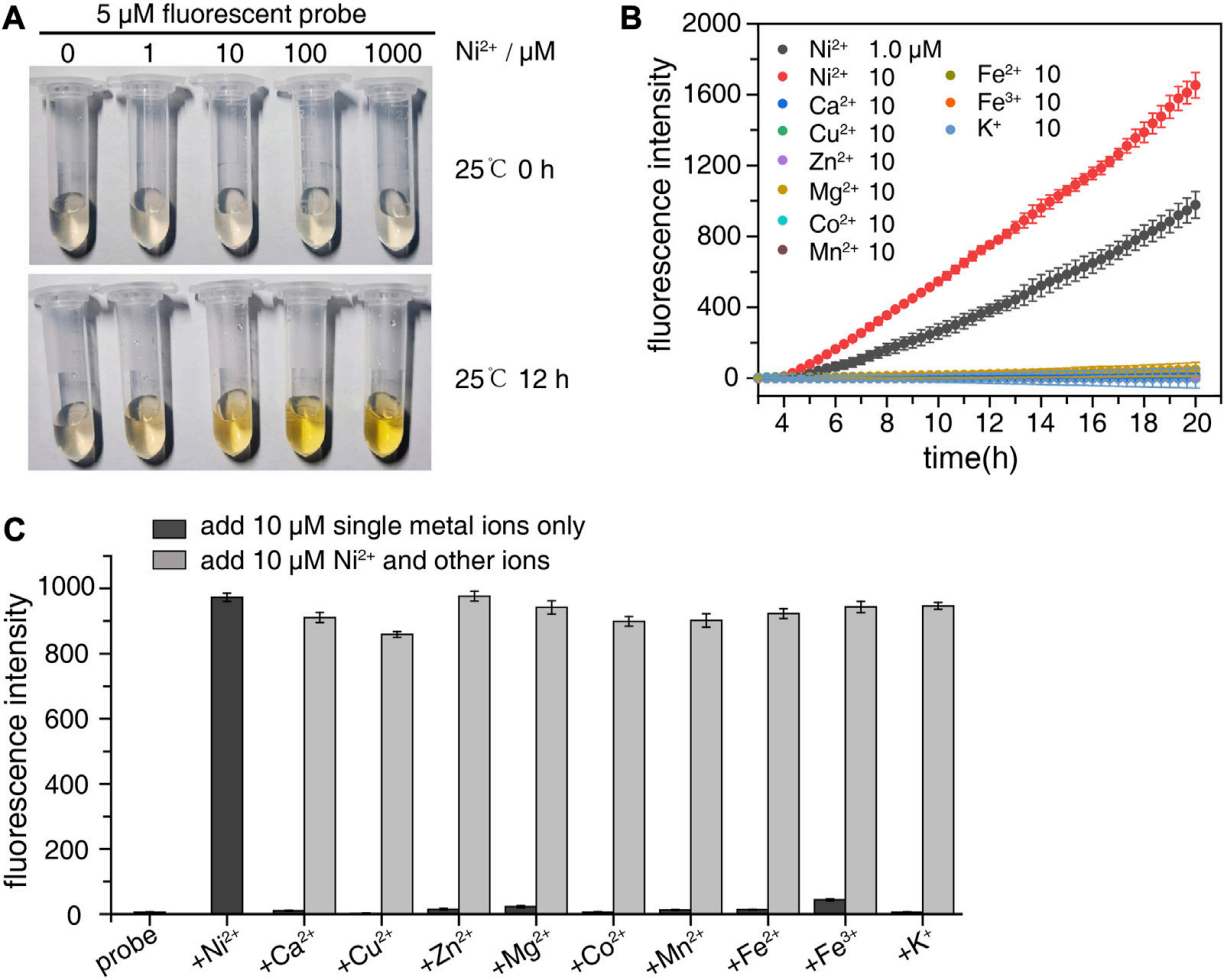
Degradation of probe SSC-Ni by nickel ions. **(A)** Nickel ions with final concentrations of 1, 10, 100, and 1,000 μM were added to the 5 μM probe SSC-Ni, respectively, and the color change of the solution was observed at room temperature. **(B)** qPCR instrument was used for real-time detection of SSC-Ni probe degradation. 1 μM and 10 μM nickel ions or 10 μM other metal ions were added to the probe SSC-Ni at a final concentration of 1 μM. The fluorescence signal of FAM was detected every 20 min at 37°C. **(C)** Study the interference effect of other metal ions on nickel ion detection. 10 μM metal ions alone or 10 μM Ni^2+^ and other metal ions were added to the probe, and the fluorescence intensity was detected after holding at 37°C for 14 h.

To study the degradation of probe SSC-Ni by nickel ions, the nickel ions with final concentrations of 1, 10, 100, and 1,000 μM were added to the 5 μM probe, respectively, and then placed at room temperature (∼25°C). A probe sample without nickel ions was used as a negative control, as shown in Figure 3A. After 12 h of reaction at room temperature, significant color changes in the solution can be observed. The higher the concentration of nickel ions, the more obvious the color change. There was no significant change in color for the negative control group. In addition, we detected the excitation and emission spectra of the probe (5 μM), as well as emission spectra after incubation at 25°C for 12 h with equivalent nickel ions (Supplementary Figure S1), and the fluorescence intensity was enhanced after the addition of nickel ions.

We also used a qPCR instrument to detect the FAM fluorescence signal of probe SSC-Ni in real-time, as shown in Figure 3B. Nickel ions at final concentrations of 1 and 10 μM were added to the 1 μM probe SSC-Ni, respectively. The fluorescence signal of FAM was detected at 37°C. As shown in the figure the fluorescence intensity gradually increases after the addition of nickel ions, and the fluorescence intensity of 10 μM increases faster than that of 1 μM nickel ions. Besides, there was no significant change in fluorescence intensity when other metal ions such as Ca^2+^, Cu^2+^, Zn^2+^, Mg^2+^, Fe^3+^, and K^+^ were added to the SSC-Ni probe. In addition, 10 μM Ni^2+^ and other metal ions were simultaneously added to the probe to monitor the fluorescence intensity, it was found that there was no significant difference between its intensity and that of 10 μM Ni^2+^ alone. This indicates that nickel ions can specifically degrade probe SSC-Ni, and the probe was not sensitive to other metal ions.

### 3.3 Preparation of standard curves

**FIGURE 4:**
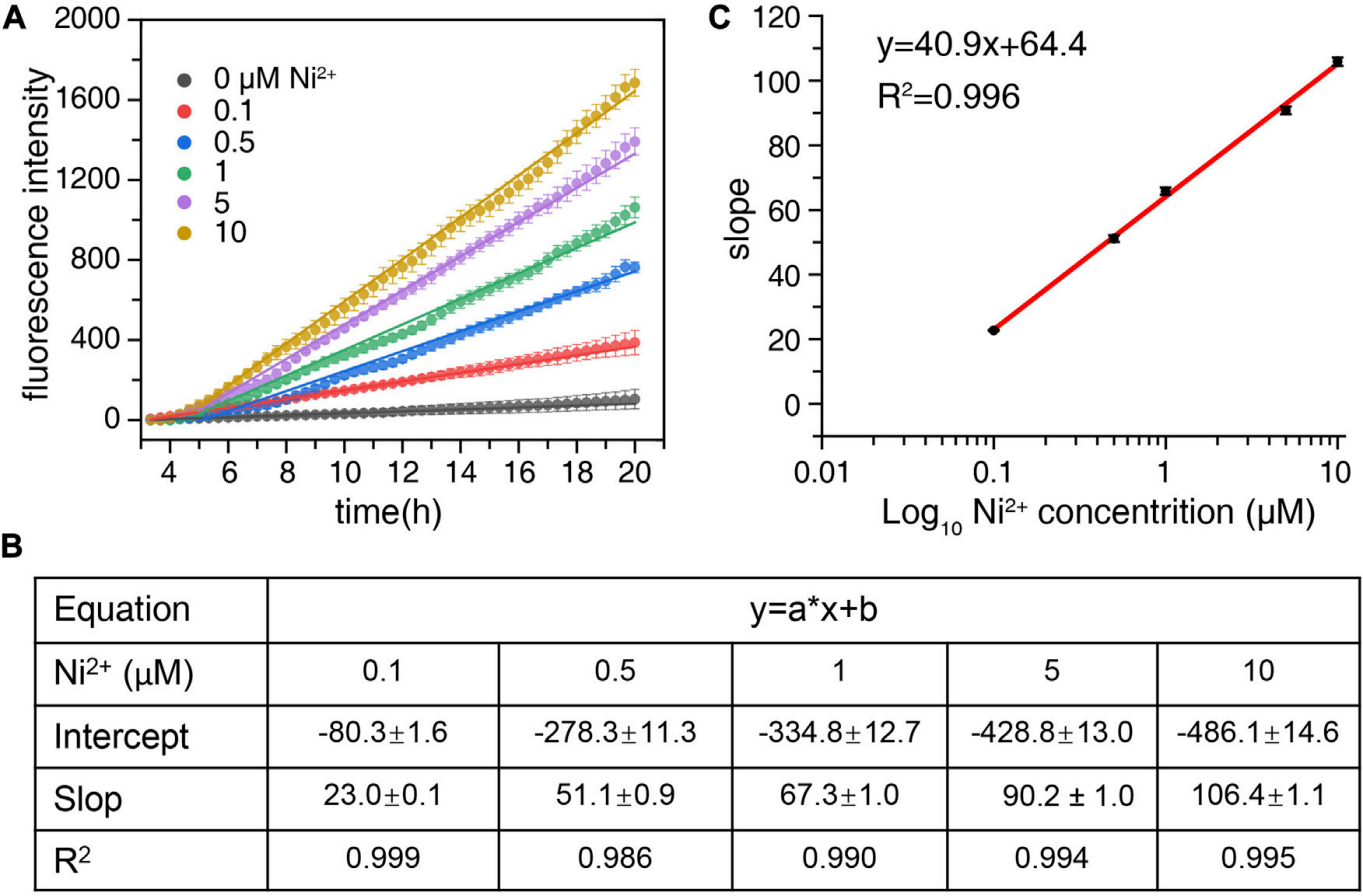
Preparation standard curve of nickel ions. **(A)** Nickel ions with final concentrations of 0, 0.1, 0.5, 1, 5, and 10 μM were added to the 1 μM probe SSC-Ni, respectively. The fluorescence signal of the FAM channel of qPCR equipment was detected at 37°C. **(B)** Origin2018 software was used for linear fitting of the fluorescence data, and related parameters such as intercept, slope, and *R* ^2^ can be obtained. **(C)** The slope of each line was plotted against log10 nickel concentration to establish a standard curve.

To further investigate the correlation between nickel ion concentration and degradation rate of probe SSC-Ni, nickel ions with different concentrations (0.1–10 μM) were added to the 1 μM probe to detect the fluorescence signal of FAM at 37°C, as shown in Figure 4A. The higher the concentration of nickel ions, the faster the fluorescence signal increases, and the fluorescence intensity data was close to a straight line. Therefore, linear fitting was performed on the fluorescence signal, and the intercept, slope, and *R* ^2^ were shown in Figure 4B, with all *R* ^2^ values above 0.986. Further, the slope of each line was plotted against log10 nickel concentration to establish a standard curve, as shown in Figure 4C. The *R* ^2^ value of the standard curve was 0.996.

The detection limit (DL) of the probe can be calculated from the formula: DL = 3 × SD/k (Yu et al., 2016). The detection limit of the SSC-Ni probe was as low as 82 nM (Figure 4C), while the relative standard deviation for 10 replicate detections of 0.1 μM Ni^2+^ was 4.8%.

### 3.4 Application of probe SSC-Ni in the blood

**FIGURE 5:**
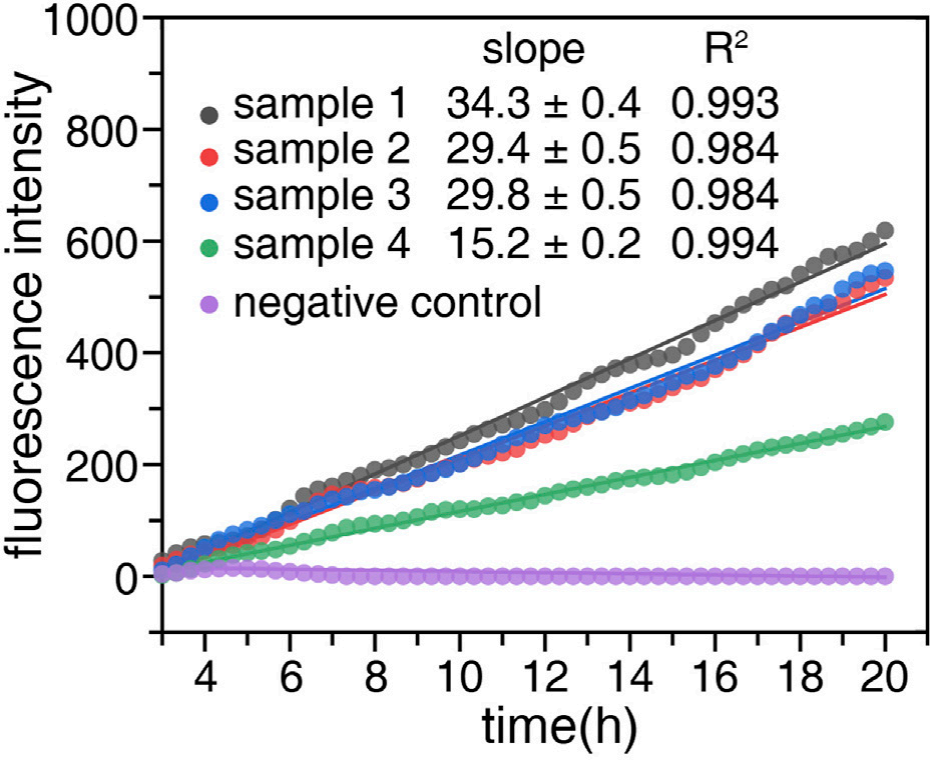
Concentration detection of nickel ions in blood samples. Four blood samples without anticoagulant were taken and serum was obtained by centrifugation at 4,000 rpm for 5 min. The probe SSC-Ni with a final concentration of 1 μM were added to serum samples one to four, and a serum sample without the probe was used as a negative control. The fluorescence signal of the FAM was detected by qPCR instrument at 37°C. Origin2018 software was used for linear fitting of the fluorescence data, and the slope and *R* ^2^ were labeled on the figure.

High concentrations of nickel ions in the human body will increase the risk of breast cancer, lung cancer, and other tumors, and this study attempts to use probe SSC-Ni to detect the concentration of nickel ions in the blood. To avoid the interference of anticoagulants on the detection, four blood samples without anticoagulants were selected. The serum was collected by centrifugation and nickel ions were added at a final concentration of 1 μM. The fluorescence signal of FAM was detected at 37°C as shown in Figure 5.

The fluorescence signal of the serum samples gradually increased with heat retention, and the fluorescence data were linearly fitted. The relevant parameters were shown in Figure 5, all *R* ^2^ values were above 0.984. The concentration of nickel ions can be calculated based on the slope and standard curve, which were 0.185 ± 0.006, 0.140 ± 0.004, 0.144 ± 0.004, 0.063 ± 0.003 μM, respectively (Table 1). Notably, the detection results were extremely close to the reported serum nickel concentration (Yu and Zhang, 2017). Then the final concentration of 1 μM and 10 μM Ni^2+^ were added to the serum samples containing the probe. The experiments were repeated for three time, and the concentration of nickel ions were shown in Table 1. The recovery rates of Ni^2+^ in serum samples were both greater than 90%.

**TABLE 1:** Detection results of nickel ions concentration in blood samples.

Blood sample	Found Ni^2+^/μM	Added/μM	Found/μM^a^	Recovery %^b^
Sample 1	0.185 ± 0.006	1	1.130 ± 0.067	94.5
		10	10.232 ± 0.120	100.5
Sample 2	0.140 ± 0.004	1	1.105 ± 0.019	96.5
		10	10.084 ± 0.166	99.4
Sample 3	0.144 ± 0.004	1	1.095 ± 0.048	95.1
		10	10.119 ± 0.031	99.8
Sample 4	0.063 ± 0.003	1	1.022 ± 0.007	95.9
		10	10.064 ± 0.099	100.0

^a^ Nickel concentrations in the blood samples were measured after the addition of 1 and 10 μM nickel (II) ion.

^b^ Recovery % was calculated by the ratio of the difference in concentration obtained from two measurements to the content of added nickel ions.

### 3.5 Comparison with other reported fluorescent probes

The SSC-Ni probe designed in this study for detecting nickel ions was compared with other reported fluorescent probes, as shown in Table 2. The detection limit and linear range of the probe are not significantly different from other reported probes. Meanwhile, the probe has high selectivity and is not susceptible to interference from other ions. There is still a significant gap in the detection capability of SSC-Ni probe compared to optical chemical sensor MMT (Aksuner et al., 2012). However, the probe designed in this study is sufficient for nickel ions detection in serum samples. In addition, the concentration of nickel ions in water samples is also within the detection range of the SSC-Ni probe (Alizadeh et al., 2016), which can theoretically detect nickel ions in water samples.

**TABLE 2:** Comparison with other fluorescent probes for nickel ion detection.

Name	DL	Linear ranges	Application	Main characteristics	References
Ra-Ni	26.2 nM	0–3 μM	water sample, living cells	a precise ratiometric fluorescent probe	Wang et al. (2022)
probe 6	0.1 μM	0–10 μM	water sample	a dual-responsive fluorescent turn-off probe	Song et al. (2020)
PAIC	2.82 μM	0–440 μM	water sample	a imidazole containing amide fluorescent turn-on probe	Annaraj et al. (2016)
compound- 3	—	0–10 μM	monitoring in EtOH solution	a fluorescent turn-off sensor comprised of pyrazoline and benzimidazole	Han et al. (2017)
probe L	1.83 μM	0–20 μM	living cells	a reversible and highly selective fluorescence “on-off-on” probe	Yu et al. (2016)
CdTe QDs	7 nM	0.01–10 μM	water sample	a CdTe QDs fluorescent turn-off-on probe	Zare et al. (2017)
MMT	0.85 nM	1.0 nM-4.4 mM	tea leave and mushroom sample	a optical chemical fluorescence quenching sensor	Aksuner et al. (2012)
SSC-Ni	82 nM	0.1–10 μM	serum and water sample	a sequence-specific cleavage fluorescent off-on probe	this study

DL: detection limit.

To the authors’ knowledge, the SSC-Ni is the only nickel ion detection probe that based on sequence-specific cleavage. The limitation of this probe is that the detection time (greater than 20 h) is much longer than other probes, which may be related to the low degradation efficiency of nickel ions on -GSHHW- peptides. Further optimization of the peptide sequences may shorten the detection time. Besides, the fluorescence intensity of the probe can be automatically measured by qPCR instruments and achieve high-throughput detection of nickel ions concentration, which can partially compensate for the long detection time.

## 4 Discussion

In conclusion, this paper introduces a novel off-on fluorescent probe called SSC-Ni for the detection of nickel (II) ion. Similar to the TaqMan probes, SSC-Ni operates on the principle of off-on fluorescence, with a lower background fluorescence signal (Yao et al., 2017). When exposed to nickel ions, the probe undergoes degradation, leading to the release of fluorescence signals, The rate of fluorescence signal enhancement is directly proportional to the concentration of nickel ions. In contrast to other probes, the SSC-Ni probe is a sequence-specific cleavage fluorescent off-on probe that detects fluorescence signals by specifically cutting the probe with nickel (II) ions. This probe exhibits high detection specificity and low detection limit (82 nM). The SSC-Ni probe is a fluorescent probe that can detect the concentration of nickel ions in the blood, which is of great value in clinical disease diagnosis.

In the practical application of the probe SSC-Ni, we also have found some defects such as the excessive detection time. This may be related to the slow degradation rate of nickel ions on polypeptide chains (Dang et al., 2019). In subsequent research, more suitable peptide chains can be screened and further optimized for the SSC-Ni probe. Moreover, the high concentration of nickel ions has a quenching effect on fluorophores (Yue et al., 2015). We have also tested for higher concentrations of nickel ions (>10 μM), and found that the fluorescence intensity of the probe could be significantly reduced due to the fluorescence quenching effect (this result was not shown), so the detection range of the SSC-Ni probe for detecting nickel ions was between 82 nM–10 μM. This detection range is coincidentally suitable for the detecting concentration of nickel ions in human blood and the environment (Kazi et al., 2008; Wang et al., 2022b).

Nickel, as a heavy metal element, poses environmental pollution risks and potential health hazards (Genchi et al., 2020). This study successfully designed and synthesized the SSC-Ni probe, which can be employed for large-scale detection of nickel ion concentration using qPCR equipment. The probe demonstrated its capability for detecting nickel ions in serum, yielding results that closely matched the reported serum nickel concentration. This finding highlights the significant clinical relevance of the SSC-Ni probe. Moreover, in theory, the probe SSC-Ni can also be applied for the detection of nickel ions in the environment.

## Data Availability

The original contributions presented in the study are included in the article/Supplementary Material, further inquiries can be directed to the corresponding authors.
