# Supplementary material for: A novel “off-on” fluorescent probe for the detection of nickel ions and its clinical application
Source: Front Bioeng Biotechnol. 2023 Sep 18;11:1261178. doi: 10.3389/fbioe.2023.1261178 (PMC10544904; doi:10.3389/fbioe.2023.1261178)
Supplement: Supplementary file 1 [file DataSheet1.DOCX]

**Supplementary materials**


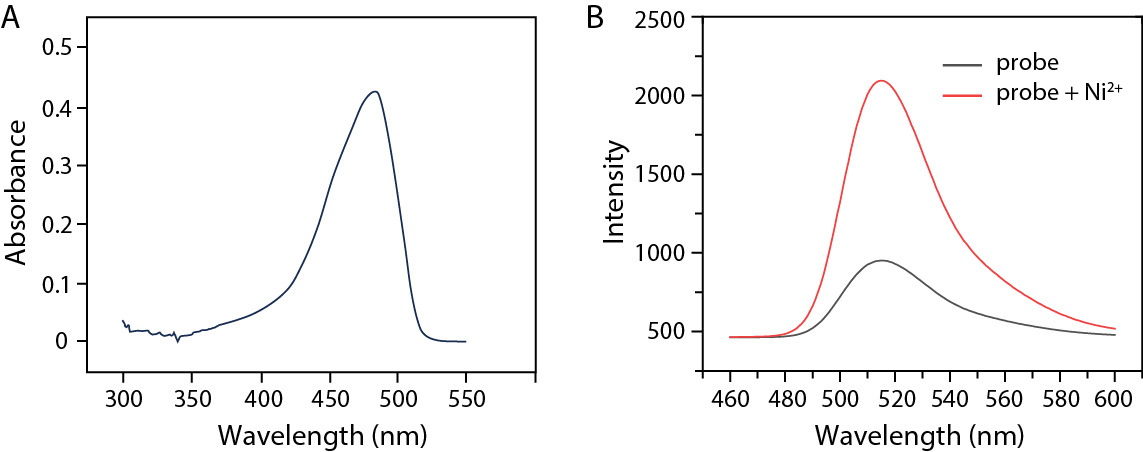


**Figure S1 Excitation and emission spectra of the probe**

A) The excitation spectra of the probe SSC-Ni (5 μM). The scanning range of the spectrum was 300-550 nm. B) The change of fluorescence intensity before and after adding 5 μM nickel ions in the test system, the probe and nickel ion were incubated at 25 ℃ for 12 h. λ_ex_ = 450 nm, the emission range was 460-600 nm, slit widths: W_ex_ = 4 nm, W_em_ = 4 nm.
